# Human antiviral B cell responses: Emerging lessons from hepatitis B and COVID‐19

**DOI:** 10.1111/imr.12953

**Published:** 2021-02-08

**Authors:** Alice R. Burton, Mala K. Maini

**Affiliations:** ^1^ Division of Infection and Immunity UCL London UK

**Keywords:** B cells, humoral immunity, memory B cell differentiation, viral infection

## Abstract

Humoral immunity is a critical component of the coordinated response required to resolve viral infections and mediate protection following pathogen clearance or vaccination. A better understanding of factors shaping the memory B cell response will allow tailored development of efficient preventative vaccines against emerging acute viral infections, therapeutic vaccines, and immunotherapies for chronic viral infections. Here, we use recent data obtained by profiling antigen‐specific B cell responses in hepatitis B as a framework to explore lessons that can be learnt from different viral infections about the diverse influences on humoral immunity. Hepatitis B provides a paradigm where successful B cell responses in resolved or vaccinated individuals can be contrasted to the failed response in chronic infection, while also exemplifying the degree to which B cell responses within infected individuals can differ to two antigens from the same virus. Drawing on studies in other human and murine infections, including emerging data from COVID‐19, we consider the influence of antigen quantity and structure on the quality of the B cell response, the role of differential CD4 help, the importance of germinal center vs extrafollicular responses and the emerging concept that responses residing in non‐lymphoid organs can participate in B cell memory.

## 1 INTRODUCTION

Antigen‐specific antiviral B cell responses are a key component in the resolution of viral infections and maintenance of immune memory following pathogen clearance or immunisation. These responses are dependent on three principal factors: (a) the generation of antibodies to clear infection; (b) the diversification and evolution of antigen‐specific responses; and (c) the persistence of long‐lived immune memory. Effective and durable humoral responses are generated in germinal center (GC) reactions, whereby B cells undergo iterative rounds of clonal expansion and somatic hypermutation to generate a diverse pool of memory B cells and plasma cells. This process is critically dependent on specialized T follicular helper cells (T_FH_) that provide vital growth and differentiation signals to GC B cells and mediate positive selection of high affinity B cell clones.

While the GC has remained the focus of B cell research, it has long been appreciated that antibody responses can also develop outside of the B cell follicle in the absence of notable GCs. Extrafollicular differentiation of naive B cells into short‐lived antibody secreting cells has been shown to mediate early antiviral immune protection in mice,^1^ with extrafollicular B cells also able to undergo affinity maturation and generate both memory and long‐lived plasma cells independently of T cell help.^2^, ^3^, ^4^, ^5^ These responses provide malleable first line defense against replicating pathogens, yet may also contribute to autoantibody production and immunopathology.

Although the roles of B cells in viral infections are diverse and wide‐ranging, including immunoregulatory cytokine production and antigen presentation, this review will concentrate on findings from new studies in hepatitis B, SARS‐CoV‐2 infection and other human and murine infections to consider emerging concepts regarding the factors that govern memory B cell differentiation and their impact on humoral immunity.

## 2 USING HBV AS A MODEL

From an immunological standpoint, Hepatitis B virus (HBV) infection provides a valuable setting to study differential B cell responses in humans, both between individuals (by comparing individuals with protective immunity to those without), and within individuals (by comparing B cell responses to different viral antigens). HBV is a DNA virus comprised of a partially double‐stranded genome packaged within a nucleocapsid of HBV core antigen (HBcAg) and enveloped by an outer shell of HBV surface antigen (HBsAg). These surface antigens are responsible for facilitating viral binding and entrance into hepatocytes and thus constitute the major antigenic epitopes of the virus and target of prophylactic vaccination. Natural HBV infection results in divergent clinical outcomes, dependent largely on the age and immune competence of the individual at the time of infection. The majority of individuals will naturally resolve infection, establishing long‐lasting immunity to the virus in the form of strong HBV‐specific T cell responses and antibodies targeting both HBcAg and HBsAg (resulting in loss of serum HBsAg, although HBV DNA typically persists as cccDNA and integrated forms in hepatocytes). However, a portion of individuals infected, particularly those infected perinatally, develop a persistent infection, where the immune response fails to control the virus and can instead trigger tissue damage leading to life‐threatening complications. While these patients do not display global defects in antibody production^6^ and maintain robust responses to HBcAg, chronic HBV infection (CHB) is characterized by an absence of detectable anti‐HBs—indeed, the diagnostic distinction of chronically infected and naturally recovered individuals is based on the persistence of HBsAg in the former. Thus, hepatitis B exemplifies the degree to which the B cell response can differ to two antigens from the same virus within an individual.

Due to the role of virus surface proteins in facilitating virus entrance, antibodies targeting HBsAg have strong neutralizing activity,^7^, ^8^ interfering with the attachment of the “a”‐determinant region of the virus to heparan sulfate proteoglycans on hepatocytes, or blocking binding of the pre‐S1 domain of HBsAg to the host cellular receptor, sodium taurocholate co‐transporting polypeptide (NTCP). Therefore, a lack of neutralizing anti‐HBs, in combination with weak and exhausted HBV‐specific CD8 T cells, is thought to contribute to viral persistence in chronically infected patients^9^, ^10^ and remains an elusive goal of therapies aiming to achieve a functional cure. Clinically, CHB can be further divided into discrete phases of disease classified on the basis of ongoing viremia, liver inflammation, and HBsAg load, facilitating detailed analysis as to how fluctuations in these disease parameters can influence immunity. Thus, divergent humoral immune responses can be studied not only between HBV‐vaccinated, resolved, or chronically infected individuals but also within patients with CHB, comparing responses to HBsAg and HBcAg and different phases of disease.

It is increasingly recognized that chronic viral infections, such as CHB, are associated with fundamental alterations in B cell populations, established through complex interactions with the immune microenvironment, including aberrant cellular interactions, persistent antigenic stimulation and inflammation. These B cell changes are reflected in dysfunctional humoral immune responses in patients with chronic infection and may represent fundamental defects critical to determining the difference between an effective or ineffective response. Questions remain surrounding how cell‐cell interactions, antigen load and the local microenvironment shape B cell immunity and function throughout the course of viral infection. Understanding these factors may reveal key insights into how B cell immunity is established following viral infection and how these pathways may be disrupted in chronic infection.

One key piece of evidence may reside in the observation that B cell depletion by anti‐CD20 or anti‐CD52 antibody therapies (eg, the use of Rituximab in the management of lymphoma) significantly increases the risk of HBV‐reactivation in HBV‐resolved patients and of viremic flares in chronically infected patients with low viral loads (HBsAg + inactive carriers).^11^, ^12^, ^13^ Long‐lived plasma cells lack expression of CD20 and so should be preserved following Rituximab treatment; as a key cell population responsible for providing long‐term antibody production, these cells might be expected to maintain serum antibody responses in the absence of memory B cells.^14^ The observation that Rituximab can lead to loss of humoral immunity in these settings therefore suggests that long‐lived plasma cells may not be reliably formed following HBV infection, instead pointing to an ongoing role for CD20‐expressing memory B cells or plasmablasts in mediating viral control. The manifestation of disease flares in chronic carriers without detectable anti‐HBs upon Rituximab may also allude to roles for B cells beyond antibodies in this setting and/or a potential contribution of antibodies not detected by standard clinical assays (eg, sequestered in immune complexes). Recent studies have identified a dominance of broadly neutralizing antibodies (bnAbs) targeting HBsAg within the HBsAg‐specific memory B cell responses in both HBV‐vaccinated and naturally resolved individuals that demonstrate protective, neutralizing potential in in vitro and in vivo models.^15^, ^16^ These bnAbs appear to play a key role in viral clearance and long‐term suppression in HBV seroconverters and thus represent a promising immunotherapeutic tool toward achieving HBV functional cure in patients with CHB.

## 3 ANTIGEN‐DRIVEN DETERMINATION OF MEMORY B CELL PROGRAMS

What can be learned from the dichotomous response to the surface and core antigens in hepatitis B about the role of antigen load or specific antigen structure in regulating humoral responses? In line with their lack of detectable antibodies against HBsAg, patients with CHB are shown to have reduced numbers of functional HBsAg‐specific responses by ELISpot compared to those with acute‐resolving infection.^17^, ^18^, ^19^, ^20^, ^21^ However, application of fluorescently labeled antigen‐bait systems was able to reveal for the first time that HBsAg‐specific B cells in fact persist at equivalent levels in patients with chronic or acute‐resolving infection and HBV‐vaccinated controls but are functionally defective, failing to produce detectable anti‐HBs upon stimulation in vitro.^22^, ^23^ Comparatively, HBcAg‐specific B cells are present at much higher frequencies than HBsAg‐specific B cells,^24^ with their number associated with elevated liver inflammation and ongoing viral replication.^25^ In stark contrast to HBsAg‐specific B cells, HBcAg‐specific B cells maintain an ability to secrete anti‐HBc antibodies upon IL‐2, IL‐21 and CD40 stimulation.^24^ These data therefore suggest that the absence of anti‐HBs in patient sera is a result of defective HBsAg‐specific B cells rather than a complete loss of this B cells with HBsAg‐ specificity.

These observations have stimulated detailed studies investigating the phenotype of B cells targeting HBsAg and HBcAg, providing insights into the differential regulation of B cell responses by antigen. Flow cytometric characterization revealed that HBsAg‐specific B cells in patients with CHB were enriched for a CD27^−^CD21^−^FcRL5^+^T‐bet^hi^ “atypical memory B cell” phenotype.^22^, ^23^ These atypical memory B cells (atMBCs)—also referred to as “tissue‐like memory B cells” and with similarities to “age‐associated B cells”—arise aberrantly in chronic infection, where they display impaired antibody‐secreting cell differentiation, antiviral effector function, and survival compared with conventional CD27^+^ memory B cells.^22^, ^26^, ^27^, ^28^, ^29^ Therefore, their presence within the HBsAg‐specific B cell compartment is consistent with these cells contributing to defective anti‐HBs responses in patients with CHB.^22^, ^23^, ^26^ Parallel analysis of HBsAg‐ and HBcAg‐specific B cells by Le Bert et al^24^, revealed that HBcAg‐specific B cells were transcriptionally distinct from HBsAg‐specific B cells, highlighting the ability of HBcAg and HBsAg to elicit distinct and dichotomous programs of differentiation and function. Unlike HBsAg‐specific B cells, HBcAg‐specific B cells were phenotypically dominated by IgG‐switched CD27^+^CD21^+^ classical memory B cells (cMBCs),^24^ which persist long‐term independently of antigen^30^, ^31^ and retain the capacity to proliferate and differentiate upon secondary exposure faster than naive B cells.^32^, ^33^ Hence, the comparative dominance of atMBCs and cMBCs in HBsAg‐ and HBcAg‐specific responses respectively may explain the difference in the formation of productive and long‐lived humoral responses.

**FIGURE 1:**
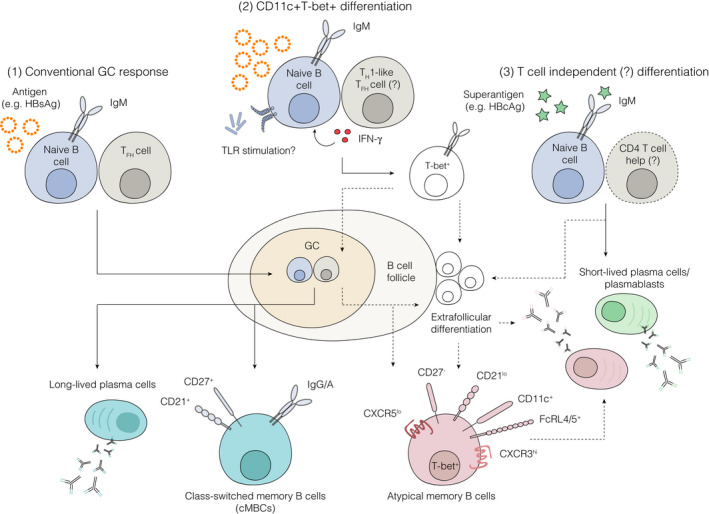
Routes to memory B cell differentiation and humoral immunity: (1) Conventional germinal center (GC) responses generate class‐switched memory B cells and long‐lived plasma cells of high affinity for antigen, as observed with anti‐HBc IgG responses in HBV‐infected patients and HBsAg‐specific B cell responses in HBV‐vaccinated individuals and patients who naturally resolve infection. Persistent antigen stimulation, combined with Type I IFN signaling and innate‐receptor sensing (eg, TLR stimulation), is hypothesized to induce T_H_1‐bias in T_FH_ responses (**2**) and drive T‐bet expression in B cells. T‐bet^+^ B cells may give rise to B cell differentiation outside of the B cell follicle or exit the GC response prematurely, establishing populations of atypical memory B cells and/or short‐lived antibody secreting cells (as observed with HBsAg‐specific responses in patients with chronic HBV infection) . In some settings (eg, anti‐HBc IgM), structural properties of the antigen may induce plasmablast responses independently of the GC, establishing short‐lived immunity (**3**)

Antigen‐experienced atMBCs have been described in many different infection settings and are thought to represent a distinct memory B cell population that diverges from cMBCs.^34^ Their presence in these contexts may give some clues as to factors that perturb memory B cell responses in chronic infections (summarized in Figure 1). Accumulating evidence suggests that atMBCs arise following repeated antigen challenge in an inflammatory setting inferring that these cells are perpetuated by persistent antigenic stimulation^35^; in support, the frequency of atMBCs observed in HIV infection falls in line with treatment‐induced reductions in viral load^36^, ^37^, ^38^ and in those that spontaneously resolve infection.^39^, ^40^ Thus, the accumulation of these cells in CHB appears to be, at least in part, driven by chronic exposure to HBV‐viral proteins, as evidenced by the higher frequency of atMBCs in patients with ongoing infection compared with vaccinated or resolved controls. In addition to secreting full replicating virions, HBV‐infected hepatocytes also release vast quantities of subviral particles—or “empty” viruses comprised of HBsAg in various sizes. These subviral particles flood the system, outnumbering infectious virus by 1000‐ to 100 000‐fold,^41^ and may be responsible for subverting functional adaptive immune responses. It therefore follows that atMBCs are enriched within the HBsAg‐specific fraction during acute and chronic infection and decline with resolution of disease. Although the frequency and phenotype of HBsAg‐specific B cells did not associate with serum measurements of HBsAg load, levels of HBsAg in the circulation are not necessarily representative of HBsAg load within the liver and can fluctuate significantly throughout the course of infection. New therapeutic approaches (including therapeutic antibodies) to substantially lower HBsAg load in patients may now reveal a role for HBsAg load in maintaining memory B cell abnormalities. Nevertheless, these data raise the possibility that atMBCs accumulate over time, suggesting that pediatric patients infected perinatally or early in life may have lower levels of atMBCs and thus a greater potential to rescue humoral immunity; how infection shapes memory B cell responses and humoral immunity at different stages in life has yet to be explored in detail and may provide important insights into the optimal time to begin antiviral treatment.

In HIV infection and CHB, T‐bet^+^ atMBCs express high levels of inhibitory receptors, including FcRL5 and PD‐1, and are refractory to BCR stimulation in vitro.^22^, ^42^ While these studies posit that atMBCs are a dysfunctional subset with impaired antiviral effector function, more recent work suggests that T‐bet^+^ B cells represent a normal component of the human immune response that expand in certain pathological settings and can be induced following prophylactic vaccination^43^, ^44^ and acute infection.^45^, ^46^, ^47^, ^48^ T‐bet‐expressing B cells detected in the circulation shortly after vaccination have been proposed to represent a pre‐plasmablast or pre‐plasma cell pool due their upregulated expression of plasma cell transcription factor Blimp‐1 and other plasma cell‐related transcripts.^43^, ^49^ Similarly, routine influenza vaccination induces a population of antigen‐specific T‐bet^+^ memory B cells^43^, ^50^ that correlate with the induction of effective humoral responses upon antigen re‐challenge,^51^ suggesting that CD11c^+^T‐bet^+^ MBCs can resemble a GC‐derived, effector‐like population able to be recalled upon secondary antigen exposure.^45^, ^51^, ^52^, ^53^ T‐bet expression in B cells has been shown in mice to drive isotype switching toward antiviral IgG2a (equivalent to IgG1 in humans),^54^ with T‐bet^+^ B cells shown to dominate gp140‐specific responses and associate with a bias of specific serum immunoglobulin toward IgG1 in patients with HIV.^49^ These studies suggest that the function and contribution of T‐bet^+^ memory B cells may differ according to context and may be restrained by inhibitory receptor expression induced following persistent antigen stimulation in settings of chronic infection. In line with this, PD‐1 blockade was shown to boost anti‐HBs^23^ and antiviral cytokine responses^22^ by HBsAg‐specific B cells in patients with CHB, in accordance with previous studies showing enhanced memory B cell proliferation and SIV‐specific antibody production following in vivo PD‐1 blockade in SIV‐infected macaques.^55^

B cell responses may also be affected where ongoing antigen production occurs alongside low‐level antibody responses. In CHB, suboptimal antibody production is thought to coalesce with high circulating levels of HBsAg, resulting in the sequestration of available antibody in the form of immune complexes.^56^ Complexed antigen may not only reduce the availability of antibodies to target the virus, but have wide‐ranging impacts on B cell function and humoral immunity through their action on Fc‐receptors.^57^ A recent study by Kardava et al,^58^ revealed that IgG3 can bind and suppress IgM‐BCR signaling in tissue‐like memory B cells from HIV‐viremic patients, with assisstance from FcγRIIB expression, complement and inflammatory components. Thus, the quantity of antigen may not only dictate memory B cell phenotype and function but sequester antibody and regulate humoral immunity through the formation of immune complexes.

Humoral immunity may also be regulated via properties intrinsic to the antigen in question. A key differential between HBcAg‐ and HBsAg‐specific responses is the ability of HBcAg to promote B cell differentiation and isotype switching independently of CD4 T cell help.^59^ HBcAg is distinct from HBsAg in its capacity to self‐assemble into highly immunogenic virus‐like particles that can bind and activate naive B cells in a T cell‐independent fashion.^60^ Due to its particulate, multivalent properties, HBcAg can crosslink membrane immunoglobulin receptors, facilitating efficient antigen presentation and co‐stimulation by HBcAg‐specific B cells to resting naive T helper cells in vivo.^61^ These structural characteristics appear to allow HBcAg‐specific B cells to bypass conventional T cell help and generate large extrafollicular antibody responses in the first instance. This capability is reflected in the kinetics of the anti‐HBc antibody response throughout the course of infection, where early detection of anti‐HBc IgM is progressively replaced by IgG isotype‐switched antibody, evoking a switch from T cell‐independent plasmablast‐dominated responses to GC‐derived immunity. Interestingly, anti‐HBc IgM can re‐appear where patients with CHB experience flares in disease and liver inflammation,^62^ possibly reflecting a burst in plasmablast responses triggered by an efflux of new HBcAg released following liver damage.

## 4 EXTRAFOLLICULAR DIFFERENTIATION VS THE GC RESPONSE

Although predominantly thought to represent a GC‐derived memory B cell population, a growing school of thought suggests that T‐bet expressing B cells (such as those seen in patients with CHB) may contribute to extrafollicular plasmablast responses under certain conditions. In SLE, extrafollicular responses are maintained by CD11c^+^ activated naive B cells that differentiate into double‐negative (DN2) B cells lacking IgD and CD27.^63^ These DN2 cells phenotypically resemble atMBCs in their high expression of T‐bet and downregulation of CXCR5 and CD21 and are epigenetically poised toward antibody‐secreting cell differentiation.^64^ As such, the expansion of CD11c^+^T‐bet^+^ B cells in CHB and other infections may be indicative of viral persistence interrupting GC formation and permitting the extrafollicular response to dominate.

Two recent studies have investigated a role for extrafollicular B cells in the immunopathology and apparent short‐lived immunity observed following severe SARS‐CoV‐2 infection.^65^, ^66^ Most COVID‐19 patients seroconvert within 7‐14 days of infection, with neutralizing antibodies that block the interaction between the “spike” glycoprotein of SARS‐CoV‐2 and its cellular receptor angiotensin‐converting enzyme 2 (ACE‐2) detected in the vast majority of recovered individuals. Strong neutralizing antibody titers and robust antigen‐specific memory B cell and circulating T_FH_ (cT_FH_) responses have been demonstrated in patients who experience a mild infection,^67^ with class‐switched memory B cells targeting the receptor‐binding domain (RBD) of the spike protein shown to persist for at least 3‐6 months post‐symptom onset.^68^, ^69^ Although titers of neutralizing antibodies are likely to wane following resolution of infection,^70^, ^71^ persisting populations of memory B cells could provide durable humoral immunity capable of diversifying in the face of a mutating virus. However, studies demonstrating disruption of GC responses during acute COVID‐19 have raised concerns that T cell‐dependent, class‐switched memory B cell differentiation may be impaired in patients experiencing severe COVID‐19 disease.^65^ Histological analysis of patients hospitalized with acute SARS‐CoV‐2 infection revealed a loss of GCs in lymph nodes and spleens compared to control samples.^65^ These observations were associated with a decreased frequency of Bcl‐6^+^ GC B cells and T_FH_ cells, together with a bias toward T‐bet^+^ T helper‐1 cells (T_H1_), extrafollicular TNF‐α production and increased frequencies of activated IgD^−^CD27^−^CXCR5^−^(CD11c^+^) DN2 B cells, recently implicated in severe cases of COVID‐19 disease.^66^ As such, although initial recruitment of DN2 B cells may play a role in providing early neutralizing responses associated with a good outcome of infection,^72^ the absence of Bcl‐6^+^ T_FH_ cells and loss of conventional GCs in the earliest stages of infection may preclude the generation of long‐lived immunity. Thus, disruption of GC responses provides a mechanistic basis that could explain the non‐durable humoral immunity^73^, ^74^, ^75^ and bias toward plasmablast responses^76^ observed in some COVID‐19 patients.

An excessive plasmablast response may also play a role in driving immunopathology in the most severe cases of COVID‐19, with plasmablast dominance in the early B cell response putatively linked to poor clinical outcome. Analysis of 125 hospitalized COVID‐19 patients revealed an immune phenotype of patients with the most severe pathology, characterized by weak induction of cT_FH_ responses and expansion of T‐bet^+^ plasmablasts (in contrast to those with better clinical outcomes where B cell responses contained T‐bet^+^ memory B cells^77^). These data suggest that T cell‐independent plasmablast responses may be responsible for the large antibody production observed in patients with severe disease.^70^, ^74^, ^78^ Similarly, anti‐HBc IgM in germline configuration has been implicated in HBV‐associated liver failure^79^ and exacerbations of disease in patients with CHB^62^ and may trigger complement‐mediated lysis leading to liver necrosis.^80^ Therefore, while plasmablast responses can provide early control during infection, excessive plasmablast‐derived antibody production following infection, alongside a delay in the emergence of the GC response, may play a key role in driving immunopathology.

The balance of the GC vs extrafollicular responses in the evolution of HBsAg‐specific memory B cell responses in CHB has not been previously studied; however, HBsAg‐specific IgG^+^ B cells from HBV‐vaccine responders, as well as patients who spontaneously resolve chronic infection, show evidence of clonal expansion and somatic hypermutation^15^, ^16^, indicative of robust GC induction where protective immunity is established. Evidence from chronic infection suggests that HBsAg‐specific B cells undergo minimal class‐switching in contrast to HBcAg‐specific B cells,^24^ although the level of somatic hypermutation within these populations has not been determined. An expansion of analogous HBsAg‐specific T‐bet^+^(CXCR5^low^CD11c^+^) B cells in CHB may therefore represent preferential activation of extrafollicular responses toward HBsAg in CHB, triggered by a lack of viral control at the infection site and GC suppression, offering a potential explanation as to the lack of long‐lived plasma cell formation and high affinity memory responses in chronic infection. However, studies in LCMV infection suggest that GC responses can operate effectively even when faced with high amounts of viral antigen, establishing a pool of high affinity antibody responses with neutralizing capacity that evolve throughout the course of chronic infection.^81^, ^82^ Comparing acute and chronic infection in parallel, Kräutler et al,^82^ showed that the repertoire and function of antibody responses diverge during the early stages of infection, culminating in long‐lasting high affinity plasma cell responses in chronic infection. It may be, therefore, that CD11c^+^T‐bet^+^ B cells differ according to context, with those arising following acute infection representing extrafollicular B cells adept at producing early antibody responses, in contrast to exhausted or dysfunctional memory B cells that persist following chronic viral infection. Understanding the reasons for this loss of long‐lived plasma cell and memory B cell formation in CHB will require architectural studies of lymphoid organs, coupled with detail analyses of cell‐cell interaction, yet may provide a mechanistic basis explaining the lack neutralizing anti‐HBs formation in CHB.

## 5 CD4 T CELL DETERMINATION OF B CELL FATE

Since GC formation and function are critically dependent on T_FH_ cell function, defective humoral immunity is closely associated with impaired CD4 T cell differentiation. The induction of broad and functional CD4 T cell responses is essential to reducing viral loads and establishing functional antibody‐mediated production. Viral persistence can be established where CD4 T cell frequencies are diminished,^83^, ^84^ with CD4 T cell dysfunction in HIV‐1 infection precluding full recovery of the B cell response upon antiviral treatment.^85^, ^86^ Early studies investigating defective anti‐HBs production in CHB suggested that persistent infection subverts protective CD4^+^ T helper responses, in turn suppressing anti‐HBs production.^19^ Co‐culture assays using allogeneic B cells from HBV‐vaccinated responders and T cells from patients with CHB failed to produce detectable anti‐HBs responses, pointing to the existence of B cell‐specific defects in combination with impaired CD4 T cell help.^17^, ^19^ Similar to CD8^+^ T cells, HBV‐specific CD4^+^ T helper cells show evidence of lymphocyte exhaustion,^87^ with decreased expression of the co‐stimulatory molecule OX40 on circulating CD4^+^ T cells associated with viral persistence.^88^ Defective T^FH^ responses towards HBsAg may be further restrained by expanded numbers of T follicular regulatory cells in chronic infection, as suggested by mouse models.^89^, ^90^ In contrast, anti‐HBc responses seem to be protected due to their ability to bypass CD4 T cell help,^59^ establishing early plasmablast responses and antibody production in germline configuration.^79^, ^80^

While T_FH_ are therefore essential for inducing T cell–dependent antibody production, the communication between CD4^+^ T cells and B cells following initial antigen stimulation is also critical to determining B cell fate. Emerging data suggest that T‐bet expression in B cells is dependent on CD4 T cell help, with adoptive transfer studies indicating that T‐bet^+^ B cells fail to form in the absence of MHC‐II or CD40‐mediated interactions.^91^ Combined with observations that IFN‐γ or IL‐21 stimulation drives T‐bet in B cells,^92^ these data suggest that T‐bet^+^ B cell generation is supported by T_H_1‐biased T cell help.

Such T_H_1‐like conditions have been shown to prevail in chronic infection and therefore may be responsible for diverting T‐bet^+^ B cell differentiation in these settings^34^, ^92^ (Figure 1). In mouse models of malaria infection, T_FH_ cells induced following infection displayed low levels of the archetypal T^FH^ markers, PD‐1 and CXCR5, instead co‐expressing T_H_1‐associated markers, T‐bet and CXCR3. This phenotype was associated with an absence of GC, which was restored upon blockade of inflammatory cytokines, TNF‐α and IFN‐γ or deletion of T‐bet,^93^ suggesting that T_H_1‐skewed T_FH_ responses may be responsible for diverting GC responses toward extrafollicular differentiation. Similarly, T_H_1‐assoicated conditions have been demonstrated to regulate expression of T‐bet and lymph node positioning receptors in B cells, resulting in the exclusion of T‐bet^+^ B cells from the GC and their accumulation in non‐GC areas of the lymph node in both patients with HIV infection and healthy controls.^94^ Here, HIV‐specific T‐bet^+^ B cells were transcriptionally distinct from those isolated HIV‐naive controls, displaying a lower frequency of somatic hypermutation relative to GC B cells and T‐bet‐ memory precursors and reduced capacity to neutralize virus in vitro.

Thus, CD4 T cells are central to determining the positioning and resultant downstream differentiation of B cell responses and are in turn differentially regulated in acute or chronic infection. This was elegantly demonstrated by De Giovanni et al,^95^ who used an adoptive transfer model to investigate differences in T_FH_ responses between acute (vesicular stomatis virus; VSV) and chronic (LCMV) infection. Early induction of Type I IFN during VSV infection promoted IL‐6 production by dendritic cells, driving T_FH_ cell differentiation and proliferation. In contrast, late exposure occurring during infection with LCMV failed to induce IL‐6, instead promoting T_H_1 cell differentiation. In vivo imaging of the GC response in this setting revealed that T_H_1‐biased CD4 T cells in LCMV infection were confined to the areas outside of the B cell follicle, providing insight into the mechanism by which CD4 T cell help may direct extrafollicular localization and differentiation of B cells.

## 6 MEMORY IN PERIPHERAL TISSUES

Differences in chemokine receptor expression by memory B cell subsets may facilitate their differential trafficking ability and accumulation outside of the lymph node and in non‐lymphoid tissues. T‐bet^+^ atMBCs and DN2 B cells have low surface expression of CXCR5 critical to lymph node homing and entry to the B cell follicle, instead expressing high levels of the inflammatory tissue‐homing marker CXCR3 and the integrin CD11c.^22^, ^54^, ^96^, ^97^ As a result, T‐bet^+^ B cells are largely absent from lymph nodes but identifiable in the blood, bone marrow, and spleen^34^ where they localize at the T:B cell border^97^ and can become resident, losing the ability to recirculate.^48^ FcRL4^+^ B cells, with many characteristics analogous of atMBCs, were described in the tonsils at much higher frequencies than that detected in the blood and bone marrow, suggesting that they may represent a specialized tissue‐based subpopulation of memory B cells.^98^ In line with this, we previously identified an expansion of CD21^−^CD27^−^T‐bet^+^CD11c^+^ memory B cells within the global and HBsAg‐specific B cell compartment in the liver relative to the blood.^22^ The frequency of intrahepatic atMBCs was further increased in HBV‐infected livers compared with healthy controls, implicating a combined effect of the liver milieu and virus in driving this expansion.

The demonstration of antigen‐specific T‐bet^+^ B cells in non‐lymphoid tissues may have important implications for local tissue‐based immunity. Memory B cells localized with tissues are likely poised to make antibody following a secondary encounter with antigen, for example influenza‐specific B cells residing in the lung rapidly differentiate in situ to provide rapid responses upon secondary infection in influenza‐infected mice.^99^ In HBV infection, local production of anti‐HBs antibodies may accelerate pathogen clearance and assist in blocking de novo infection of hepatocytes or promoting ADCC‐mediated elimination of infected cells.^10^

It is also plausible that tissue‐localized B cells may be capable of participating in ectopic GC reactions. It has long been described that tertiary lymphoid structures resembling GCs can arise in non‐lymphoid tissues and assist in the activation, proliferation, and differentiation of B cell responses. The formation of ectopic GCs is dependent on the preparation and remodeling of peripheral tissues into one reminiscent of secondary lymphoid organs (SLO) capable of supporting GC responses. Viral infection has been clearly shown to support lung remodeling following influenza infection: Type I IFN produced in response to infection rapidly induces CXCL13, in turn driving B cell recruitment and lung remodeling to facilitate the formation of ectopic GCs.^100^ These responses can have profound benefits for local immunity; for example, the development of ectopic GCs in the lung following Influenza‐A infection is sufficient to prime antigen‐specific T and B cell responses independently of SLO‐derived responses to protect against secondary infection^101^, ^102^ and can generate memory B cells and plasma cells with greater cross‐reactive potential to enhance protection against highly mutating viruses.^103^

Intrahepatic lymphoid follicles (ILFs) with features of ectopic GCs (including Bcl‐6 and Ki67) are well‐documented in Hepatitis C virus (HCV) infection^104^ and are postulated to facilitate expansion of both HCV‐specific and autoreactive clones.^105^, ^106^ In comparison, B and T cell aggregates in patients with CHB have been shown to lack a well‐formed GC,^105^ suggesting that an absence of mature tissue‐based responses may contribute to overall impaired humoral immunity in CHB. A recent paper testing the immunotherapeutic effects of TLR‐7 agonists in HBV‐infected chimpanzees demonstrated a transient induction of these aggregates coinciding with prolonged suppression of serum viral DNA and antigens. Although preliminary investigation suggested that these structures did not contain GC reactions, the association between the formation of these structures and antiviral response to treatment suggested that they may play a role in promoting an effective response against HBV.^107^ Further in‐depth analysis is required to investigate whether memory B cells can become activated and differentiate within the liver or traffic to the liver post‐activation, and how this affects the development and maintenance of local humoral responses.

Whether intrahepatic B cells represent a transient population circulating through the highly vascularized liver, or a pool of resident B cells capable of persisting in the liver long‐term is not currently known. A growing body of evidence suggests that viral infection may seed populations of tissue‐resident B cells with the capacity to remain within peripheral tissues and provide lasting protection against secondary infection. Influenza‐specific B cells have been demonstrated to accumulate in the lung where they can persist for up to five months following resolution of infection and secrete high levels of neutralizing antibodies in the local microenvironment.^108^ As with tissue‐resident T cells, emerging data suggest that B cells responding to infection at a peripheral site are imprinted by local antigen encounter within the lung, allowing them to acquire homing capabilities that facilitate their in situ differentiation and retention within the tissue shortly after infection.^99^ Mice with enriched influenza‐specific tissue‐resident B cell populations (B_RM_) in the lung responded more rapidly than mice with memory B cells at systemic sites due to the rapid differentiation of B_RM_ into antibody‐producing plasma cells directly at the site of infection.^99^ The selection of B cells that seed these tissue‐resident compartments might therefore determine the specificity and affinity of local responses to viral antigens during secondary responses and may explain the broad reactivity of ectopic GC responses observed.^103^, ^109^

## 7 OUTLOOK

To date, the study of humoral immunity to viral infections has predominantly focused on antibodies rather than the cells responsible for producing these effector molecules. Following the resolution of an acute infection like COVID‐19, antibodies may wane in some individuals over time, underscoring the importance of determining whether memory B cells of the correct specificity persist to allow a rapid response upon re‐infection. In a chronic infection like CHB where anti‐HBs antibodies are undetectable, identification of persistent HBsAg‐specific memory B cells has raised the possibility that endogenous humoral immunity could be revived by identifying the defects limiting their function. Thus, recent studies using fluorescent baits to characterize antigen‐specific B cell responses from patients with acute infections like COVID‐19 and chronic infections like HBV have started to expand our understanding of the complexity of memory B cell responses. Flow cytometric analysis has allowed their ex vivo quantification and phenotypic characterization, revealing memory subsets with different isotype expression, homing patterns, and signatures of T_FH_ interactions and GC reactions, as well as transcriptional factors determining antiviral potential such as T‐bet.

However, the level of characterization of memory B cells still lags far behind that of T cells in viral infections and much remains to be learnt. In addition to targeted phenotypic analysis, unbiased profiling combining RNA‐sequencing with B cell receptor analysis will allow more comprehensive characterization of memory B cell responses. Analysis of B cells specific for different viral antigens, carried out in tandem with antigen‐specific T_FH_ responses and focused analysis of pathways such as CD40/CD40‐L, will help to determine their potential for productive interaction. Longitudinal analyses during dynamic phases of infection will clarify their temporal kinetics, as exemplified by the recent demonstration that spike‐specific memory B cells continue to increase in frequency in the 6 months following COVID‐19 infection.^68^ Where possible, parallel analyses of memory B cell and plasma cell responses in lymph nodes, gut and bone marrow will give a better understanding of how well circulating frequencies and phenotypes reflect humoral antiviral immunity sequestered in these compartments. Similarly, our demonstration that HBsAg‐specific B cells can be identified in human liver^22^ reinforces the need for more in‐depth studies of memory B cells compartmentalized in the liver and in other settings of virally infected non‐lymphoid organs such as the lungs in COVID‐19.

## CONFLICT OF INTEREST

MKM has received collaborative research funding from Gilead Sciences, F. Hoffmann‐La Roche and Immunocore and has served as a consultant or on advisory boards for Gilead Sciences, F. Hoffmann‐La Roche, Immunocore and GSK. MKM and ARB have filed IP on a method to enhance HBV‐specific immune responses.
